# Overexpression of the CC-type glutaredoxin, *OsGRX6* affects hormone and nitrogen status in rice plants

**DOI:** 10.3389/fpls.2015.00934

**Published:** 2015-11-03

**Authors:** Ashraf El-Kereamy, Yong-Mei Bi, Kashif Mahmood, Kosala Ranathunge, Mahmoud W. Yaish, Eiji Nambara, Steven J. Rothstein

**Affiliations:** ^1^Department of Molecular and Cellular Biology, University of GuelphGuelph, ON, Canada; ^2^Division of Agriculture and Natural Resources, University of California Cooperative Extension Kern CountyBakersfield, CA, USA; ^3^Department of Biology, College of Science, Sultan Qaboos UniversityMuscat, Oman; ^4^Department of Cell and Systems Biology, University of TorontoToronto, ON, Canada

**Keywords:** rice, glutaredoxin, CC-type, nitrogen, cytokinin, GA

## Abstract

Glutaredoxins (GRXs) are small glutathione dependent oxidoreductases that belong to the Thioredoxin (TRX) superfamily and catalyze the reduction of disulfide bonds of their substrate proteins. Plant GRXs include three different groups based on the motif sequence, namely CPYC, CGFS, and CC-type proteins. The rice CC-type proteins, *OsGRX6* was identified during the screening for genes whose expression changes depending on the level of available nitrate. Overexpression of *OsGRX6* in rice displayed a semi-dwarf phenotype. The *OsGRX6* overexpressors contain a higher nitrogen content than the wild type, indicating that OsGRX6 plays a role in homeostatic regulation of nitrogen use. Consistent with this, *OsGRX6* overexpressors displayed delayed chlorophyll degradation and senescence compared to the wild type plants. To examine if the growth defect of these transgenic lines attribute to disturbed plant hormone actions, plant hormone levels were measured. The levels of two cytokinins (CKs), 2-isopentenyladenine and trans-zeatin, and gibberellin A1 (GA1) were increased in these lines. We also found that these transgenic lines were less sensitive to exogenously applied GA, suggesting that the increase in GA1 is a result of the feedback regulation. These data suggest that OsGRX6 affects hormone signaling and nitrogen status in rice plants.

## Introduction

Glutaredoxin (GRXs) are small glutathione dependent oxidoreductases that belong to the Thioredoxin (TRX) superfamily and catalyze the reduction of disulfide bonds of their substrate proteins in the presence of glutathione. GRXs perform their functions through two different mechanisms depending on the structure of the motif that contains the cysteine sulfhydryl group. The dithiol reaction requires the presence of two conserved terminal cysteines in the motif (CXXC) while in the monothiol reaction the target protein is reduced only through one N-terminal cysteine that can be a part of the CXXC or CXXS motif. Both reduction reactions result in a reversible posttranslational and structural modification of the target proteins (Xing et al., 2006). The plant glutaredoxins have been shown to be involved during various stress responses including Type II peroxiredoxins reduction, dehydroascorbate reduction, peroxidase activity, methylviologen response, methionine sulfoxide reductase regeneration and interaction with thioredoxins. Other functions include the reduction of ribonucleotides, Calvin, and Krebs cycle regulation, regulation of the signaling pathway and [Fe-S] and haem assembly (Rouhier et al., 2006, 2008; Meyer et al., 2008, 2009). Moreover, overexpression of some members of plant glutaredoxin regulate the plant responses to stress conditions. For example the *Pteris vittata* glutaredoxin PvGrx5 when overexpressed decreases arsenic accumulation in *Arabidopsis* leaves and increases the tolerance to arsenic and high temperature (Sundaram et al., 2009; Sundaram and Rathinasabapathi, 2010). The tomato glutaredoxin gene *SlGRX1* has also been reported to regulate plant responses to oxidative stress. Transgenic plants overexpressing *SlGRX1* exhibited increasing tolerance to hydrogen peroxide, drought and salt stress (Guo et al., 2010). Plant GRXs include three different groups based on the motif sequence, CPYC, CGFS, and CC-type proteins (Ziemann et al., 2009) and a fourth group was added in rice containing the GRL-type (Garg et al., 2010). The CC-type GRXs proteins possess distinctive CC (M/L) (C/S) conserved active sites in *Arabidopsis*, while this motif is extended to C(C/G/F/Y/P) (M/L) (C/S/I/A) in rice GRX proteins (Ziemann et al., 2009). For instance, the *Arabidopsis* ROXY1 and ROXY2 mutants are the first plant GRXs phenotypes which revealed the requirement for these genes in petal and anther development (Xing et al., 2005; Xing and Zachgo, 2008). The rice orthologues *OsROXY1* and *OsROXY2* have been shown to mediate petal morphogenesis when expressed in *Arabidopsis* (Wang et al., 2009). Further, overexpression of the *Arabidopsis* SA induced *ROXY19* down-regulated the transcription of the jasmonic acid dependent plant defensin gene (PDF1.2; Ndamukong et al., 2007). Although the rice genome contains 48 genes encoding GRX proteins (Garg et al., 2010), only a few of them have been cloned and characterized (Minakuchi et al., 1994). The rice *OsROXY1* and *OsROXY2* have been characterized and shown to affect flower development in *Arabidopsis* (Wang et al., 2009). Sharma et al. (2013), reported the involvement of the rice *OsGRX8* in the plant responses to Auxin, salinity, osmotic, and oxidative stress. Further, overexpression of the rice *OsGRXC2;2* increased yeast tolerance to menadione and the tolerance of rice green leaves to methyl viologen, suggesting the involvement of *OsGRXC2;2* in the defense against oxidative stress (Morita et al., 2015).

Expression analysis of the glutaredoxin suggests their role in regulating plant growth and development throughout the plant life cycle and in addition their expression is induced by several environmental stimuli including hormones and different stress conditions (Garg et al., 2010). Glutaredoxins have been suggested to play a role in the redox signaling pathway in different organisms (Fujino et al., 2006). Plant hormones play a vital role during the different life cycle stages and consequently affecting hormone biosynthesis or signaling pathways alters plant morphology and development. Cytokinins (CK) and gibberellins (GA) are plant hormones that influence cereal yield (Ashikari et al., 2005) and are required for plant development (Santner et al., 2009). Glutaredoxins have been reported, to be involved in plants responses to hormones such as auxin and ethylene (Zander et al., 2012; Sharma et al., 2013) and involved also in fungal infection (La Camera et al., 2011). Alteration of the components involved either in the signaling or the biosynthesis of either of these hormones affects plant development. For example, overexpression of the rice CK Type-A Response Regulator gene *OsRR6* resulted in dwarf phenotypes with poorly developed root systems and panicles (Hirose et al., 2007). This phenotype was associated with an increase in the CK content and alteration in the expression of genes encoding CK oxidase/dehydrogenase. Further, overexpression of the rice *OsRR3* and *OsRR5* genes reduced plant sensitivity to exogenous CK (Cheng et al., 2010).

The genetic modification of GA biosynthesis or signaling pathways negatively affects plant size and seed development (Ueguchi-Tanaka et al., 2005; Yamamoto et al., 2010). Further, overexpression of the GA negative regulator *SLR1* in rice reduced the growth and development of transgenic plants (Itoh et al., 2005). Besides the role of GA and CK in plant development, these hormones also regulate nutrient transport and metabolism. Increase in nutrient transport and metabolism evidenced in transgenic rice plants overexpressing *OsRR6* was associated with alteration in the CK signaling pathway (Hirose et al., 2007). Sykorová et al. (2008) reported that overexpression of the CK biosynthesis gene *IPT* in wheat increased CK content, nitrate influx and nitrate reductase activity. Although, there is clearly an interaction between hormone action and nutrient acquisition and metabolism pathways, there has been no published evidence of the involvement of glutaredoxin in these processes.

The CC-type class proteins are the most functionally evolved and are involved in diverse biological processes. Among these CC-type proteins, *OsGRX6* expression is induced by the plant hormones IAA, ABA and SA and at the same time is responsive to methyl viologen, hydrogen peroxide, desiccation, salt, and arsenate stress (Garg et al., 2010). However, to date, functional validation and characterization of the members of the OsGRX have not been elucidated. In the present study, we describe the functional analysis of the *OSGRX6*, a CC-type GRXs gene that was identified by Bi et al. (2009), during the screening for genes involved in nitrogen use efficiency (NUE) in rice.

## Materials and methods

### Plant materials and growth condition

Rice seeds (*Oryza sativa* L. Kaybonnet) were planted in pots containing 75% vermiculite and 25% peat moss (SunGro Horticulture Canada Ltd., BC, Canada). Plants were grown in a full nutrient condition by adding 1 g of the slow release fertilizer that contained N-P-K, 13-13-13 supplemented with micronutrients (Plant Products Co. Ltd, ON, Canada) to each 500 ml pot. Plants were grown under growth cabinet (Conviron, Manitoba, Canada) conditions consisted of 12 h light (~500 μmol m^−2^s^−1^) at 29°C and 12 h dark at 23°C and 65% relative humidity.

### Constructs preparation and plant transformation

The constructs for overexpressing OsGRX6 (Os01g0667900) were created using the maize ubiquitin promoter. Agrobacterium-mediated transformation was used to generate the transgenic plants as described in Bi et al. (2009). The positive transformed plants were selected by the Phosphomannose isomerase (PMI) test (Negrotto et al., 2000).

### Measurement of cytokinin (CK) and gibberellin (GA) contents

Leaves of 4 weeks old rice plants were used for quantitative analysis of the CK and GA using the methods described by Preston et al. (2009) as follow. Plant hormones were extracted from 0.5 to 1 g of tissues using solid phase extraction as described by Preston et al. (2009) with minor modifications. Purified fractions were analyzed by a liquid chromatography-electronspray ionization-tandem mass spectrometry (LC-ESI-MS/MS) as described by Toh et al. (2012). Briefly, freeze dried tissues were homogenized with 80% (v/v) methanol containing 1% (v/v) glacial acetic acid in a TissueLyser II (Qiagen), added 500 pg d_2_-GA_1_, 200 pg of d_5_-tZ and 100 pg of d_6_-2iP, and stored at 4°C overnight. The samples were centrifuged to remove debris, and the pellet was washed twice. The supernatant was evaporated in a Speed Vac, reconstituted in 1 mL of 1% (v/v) acetic acid, and passed through pre-equilibrated Oasis HLB column (Waters) according to the manufacture's instruction. The CK and GA fractions were eluted with 1 mL methanol containing 1% (v/v) acetic acid after washing with 1 mL of water containing 1% (v/v) acetic acid, evaporated in a Speed Vac, reconstituted in 1 mL of water containing 1% (v/v) acetic acid. The resultants were applied to preconditioned Oasis MCX columns (Waters), and washed with 1 mL of water containing 1% acetic acid. The GA_1_ fraction was eluted with 1 mL of methanol containing 1% acetic acid and the CK fraction was eluted with 1 mL of 60% methanol containing 5% (v/v) ammonia. The solvent was removed under vacuum and subjected to the LC-ESI-MS/MS (Agilent 6410 TripleQuad LC/MS system). An LC (Agilent 1200 series) equipped with a 50 × 2.1 mm, 1.8-μm Zorbax SB-Phenyl column (Agilent) was used with a binary solvent system comprising 0.01% (v/v) acetic acid in water (Solvent A) and 0.05% (v/v) acetic acid in acetonitrile (Solvent B). Separations were performed using a gradient of increasing acetonitrile content with a flow rate of 0.2 mL min^−1^. The gradient was increased linearly from 3 to 50% B over 15 min. The retention time of tZ, 2iP, and GA_1_ were 10.0, 12.8, and 11.4 min. MS/MS conditions were as follows: capillary 4.0 kV; source temperature, 100°C; desolvation temperature, 350°C; cone gas flow, 0 L/min; desolvation gas flow, 12 L/min; fragmentor, 110 for cytokinins and 150 for GA_1_; collision energy, 16 for tZ, 14 for 2iP, and 24 for GA_1_; polarity, positive for cytokinins and negative for GA_1_; MS/MS transition, 225/137 and 225/136 m/z for d_5_-tZ and 220/136 m/z for endogenous tZ, 210/137 m/z for d_6_-2iP and 204/136 m/z for endogenous 2iP, 349/261 m/z for d_2_-GA_1_ and 347/259 for endogenous GA_1_. A calibration curve was made using MassHunter software.

### Determination of cell dimensions of the sheath

To detect the cell dimensions of the mid cortical cells of the sheath, freehand, longitudinal sections were made from the middle of the leaf sheath of wild type and transgenic (OE-GRX6-1 and OE-GRX6-2) rice plants grown in soil for 4 weeks. The samples were then immersed completely in 2% (w/v) NaOH and incubated at 60°C for 36 h until cells were visible (Peterson and Lefcourt, 1990). After incubation, samples were rinsed three-times with distilled water and stained with 0.05% (w/v) Toludine blue O in phosphate buffer. Stained sections were then observed with transmitted white light (TBO; O'Brien et al., 1964) under a light microscope (Leica DMLS2, Leica, Wetzlar, Germany). Fifteen longitudinal sections from three plants were used to determine the cell size of each genotype.

### Measurement of total chlorophyll content and photochemical quantum yield of photosystem II (PS II) of leaves

Chlorophyll was extracted with 80% acetone from 0.1 g samples of frozen leaves of wild type and OsGRX6 transgenic plants. The extraction was repeated several times until the tissue was white. The extract was measured spectrophotometrically at 645 and 663 nm. Chlorophyll contents were determined according to the method of Arnon (1949).

The photochemical quantum yield of Photosystem II [PS II; $Y\left(II\right)=F_{M}^{\prime }$ - $F^{\prime }∕F_{M}^{\prime }$; (Genty et al., 1989)] of 30-d-old wild type and transgenic (OE-GRX6-1 and OE-GRX6-2) plants was measured using Pulse-Amplitude Modulated (PAM) photometry using a PAM fluorometer (H. Walz, Effeltrich, Germany). Here, *F*′_*M*_ is maximal fluorescence yield when Photosystem II reaction centers are closed by a strong light pulse (relative units), whereas *F*′ is fluorescence yield shortly before onset of a strong light pulse (relative units). Readings were taken in the same growth chamber where plants were grown. Fully-expanded third and fourth leaves from the top were used for the measurement. During measurement, the readings were taken at inter-venial areas, avoiding veins of the leaf blade. Three repeated measurements were taken from each leaf and two leaves were selected from each plant. Ten plants were used from each genotype for the measurements.

### Quantitative real-time PCR analysis

Quantitative real-time PCR was carried out using specific primers designed from the sequence of the chosen genes. Total RNA was isolated from plant tissues using TRI-Reagent (Sigma-Aldrich, MO, USA). To eliminate any residual genomic DNA, total RNA was treated with RQ1 RNase-free DNase (Promega, WI, USA). cDNA was synthesized from total RNA by using the Reverse Transcription System kit (Quanta, MD, USA). Quantitative real-time PCR was carried out in an ABI 7300 system (Applied Biosystems) using PerfectCTa®SYBR Green super mix (Quanta Biosciences) according to the manufacture instruction. Primer Express 2.0 software (Applied Biosystems, CA, USA) was used to design the primers for the target genes (Table 1S). Relative quantification (RQ) values for each target gene relative to the internal control actin were calculated by the 2CT method (Livak and Schmittgen, 2001).

### Hormonal treatments

Seeds of the wild type and the transgenic lines were incubated with a nutrient solution containing the indicated concentration of GA_3_ and/or 6-Benzylaminopurine (BAP) as a source of gibberellin and cytokinin, respectively.

### Preparation of protein extracts and SLR1 detection

Tissues of the 1-week old seedlings were used for protein extraction. After grinding the tissues in liquid nitrogen, total proteins were extracted in five volume of the extraction buffer contains [20 mM Tris-HCl, pH 7.5, 150 mM NaCl, 0.5% Tween-20, 1 mM EDTA, 1 mM dithiothreitol (DTT)] containing 1/200 volume of the complete protease inhibitor cocktail (Sigma, Aldrich, CA). After extraction and protein quantification, an equal protein amount was mixed with 2x sample buffer, boiled for 5 min and separated by 8% SDS-PAGE and transferred to a Hybond ECL membrane (Amersham Pharmacia Biotech) by semi dry blotting. The blots were incubated with the SLR1 antibody and the signal was detected as described by Ueguchi-Tanaka et al. (2005).

### Total nitrogen analysis

Total nitrogen was determined in the samples based on the Dumas Method (Dumas, 1831). Samples were dried, and ground or sieved prior to analysis. The samples were combusted in a sealed system. Nitrogen compounds released were reduced to N_2_ gas, which was measured by a thermal conductivity cell using the LECO FP428.

### Statistical analysis

All statistical analyses were performed using SigmaStat (SPSS Inc., Chicago, IL) with an error rate set at α = 0.05. The significance difference between treatments was tested using Tukey's Honestly Significant Difference Test.

## Results

### Overexpression of *OsGRX6* which encodes a CC-type glutaredoxin affects plant development

Sequence analysis of the OsGRX6 revealed the presence of a GSH binding site known to be present in glutaredoxin proteins (Figure S1A). Analysis of the amino-acid sequence revealed that the OsGRX6 protein contains a “CCLI” motif (Figure S1B).

**Figure 1:**
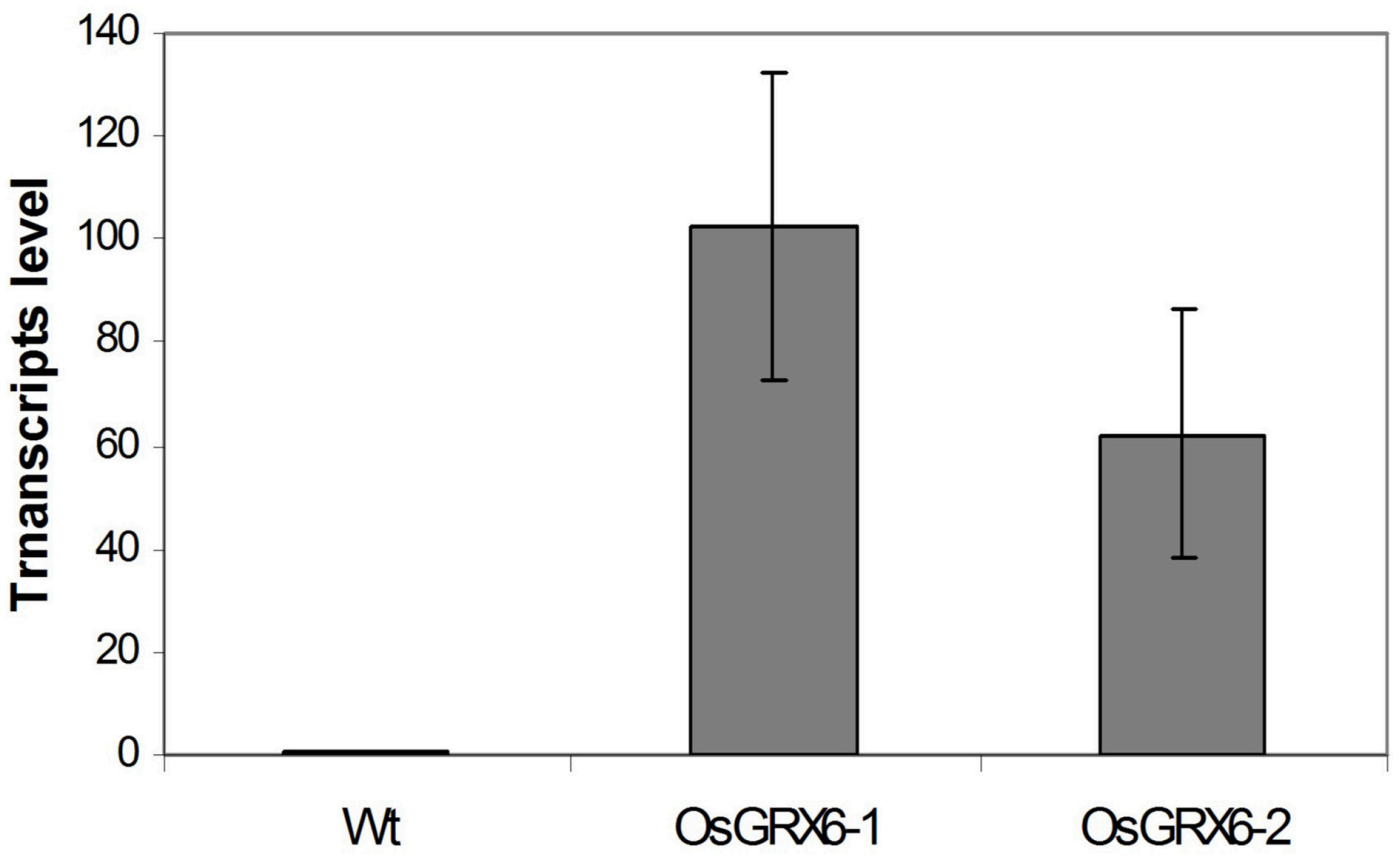
**OsGRX6 transcripts level in the leaves of 4 weeks old transgenic rice plants relative to wild type plants**. Values are the mean of three biological and three technical replicates (±SD).

**Figure 2:**
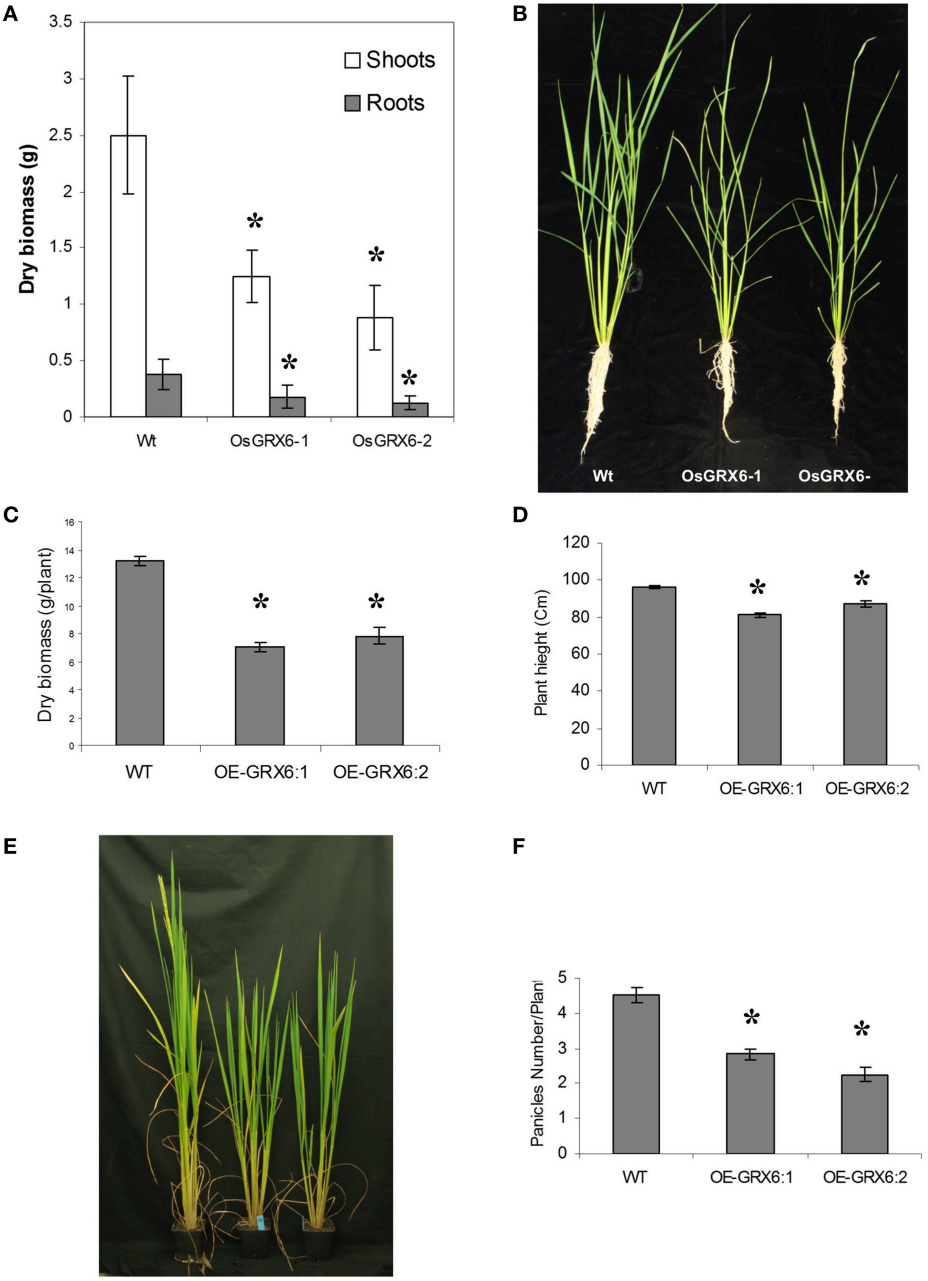
**OsGRX6 overexpression alters the rice plant morphology and development**. Shoots and roots morphology and dry biomass at 4 weeks **(A,B)**, at harvest **(C,D)**, the delaying in flowering time **(E)** and the lower panicles number in the transgenic plants compared to the wild-type plants **(F)**. Data are means ±SD of 24 plants, Asterisks indicate significant differences at *P* ≤ 0.05.

In the present study, rice plants that overexpressed *OsGRX6* under the control of maize ubiquitin promoter showed markedly higher level of its transcript (Figure 1). Ten individual transgenic lines were created and analyzed. Similar phenotypes were observed for these lines and data from two of these selected lines were chosen to be presented herein. Overexpression of the *OsGRX6* was associated with a delay in plant growth and flowering time compared to the wild type plants. The transgenic plants were smaller than the wild type and therefore had less vegetative and root dry biomass. This phenotype was recorded after 4 weeks from planting (Figures 2A,B) and at the harvesting stage (Figures 2C,D). Moreover, transgenic plants showed a delay in flowering time and decreased panicle number (Figures 2E,F).

### Overexpression of *OsGRX6* increases grain weight

**Figure 3:**
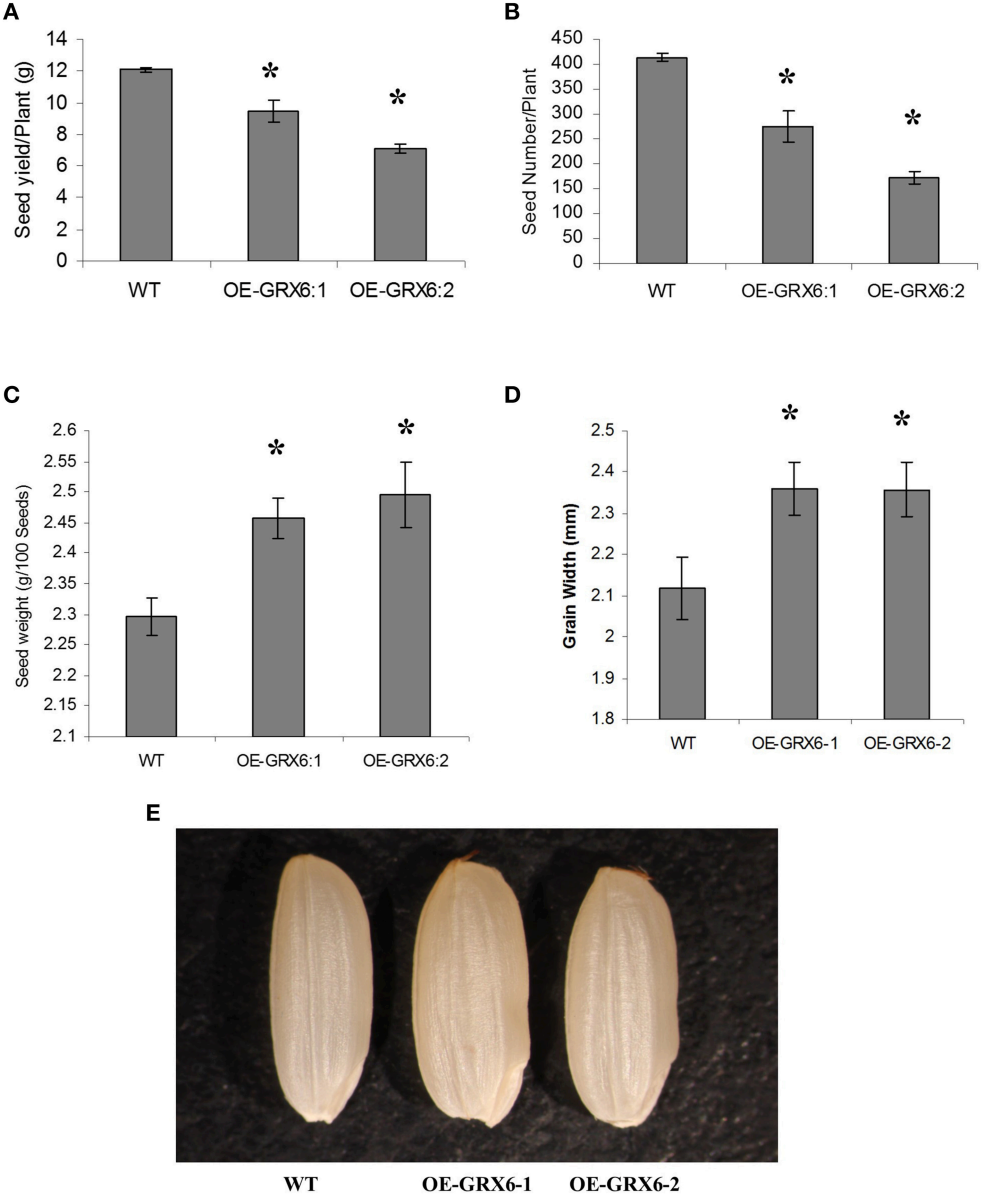
**Rice grains yield components as affected by the overexpression of the OsGRX6**. Total seed weight per plant **(A)**, seeds number **(B)**, Data are means ±SD of 24 plants, individual seed weight **(C)**, Data are means ±SD of six replicates, and grain width **(D,E)**, Data are means ±SD of six replicates of 20 grains. Asterisks indicate significant differences at *P* ≤ 0.05.

Although, the overexpression of *OsGRX6* reduced grain yield including seed yield and seed number per plant (Figures 3A,B), a significant increase in the weight of grains was observed in the transgenic plants compared to the seeds of wild type plants (Figure 3C). This increase in grain weight was 7 and 8.6% in the two independent overexpression lines. In addition there was an increase in the grain width which was 11.5 and 11.4% higher in the two overexpression lines compared to wild-type (Figures 3D,E). Grain size measurements revealed no significant changes in the grain length, although a significant increase in the grain width was observed in the transgenic lines compared to the wild-type seeds (Figures 3D,E).

### OsGRX6 alters nitrogen content in rice plants and seeds

**Figure 4:**
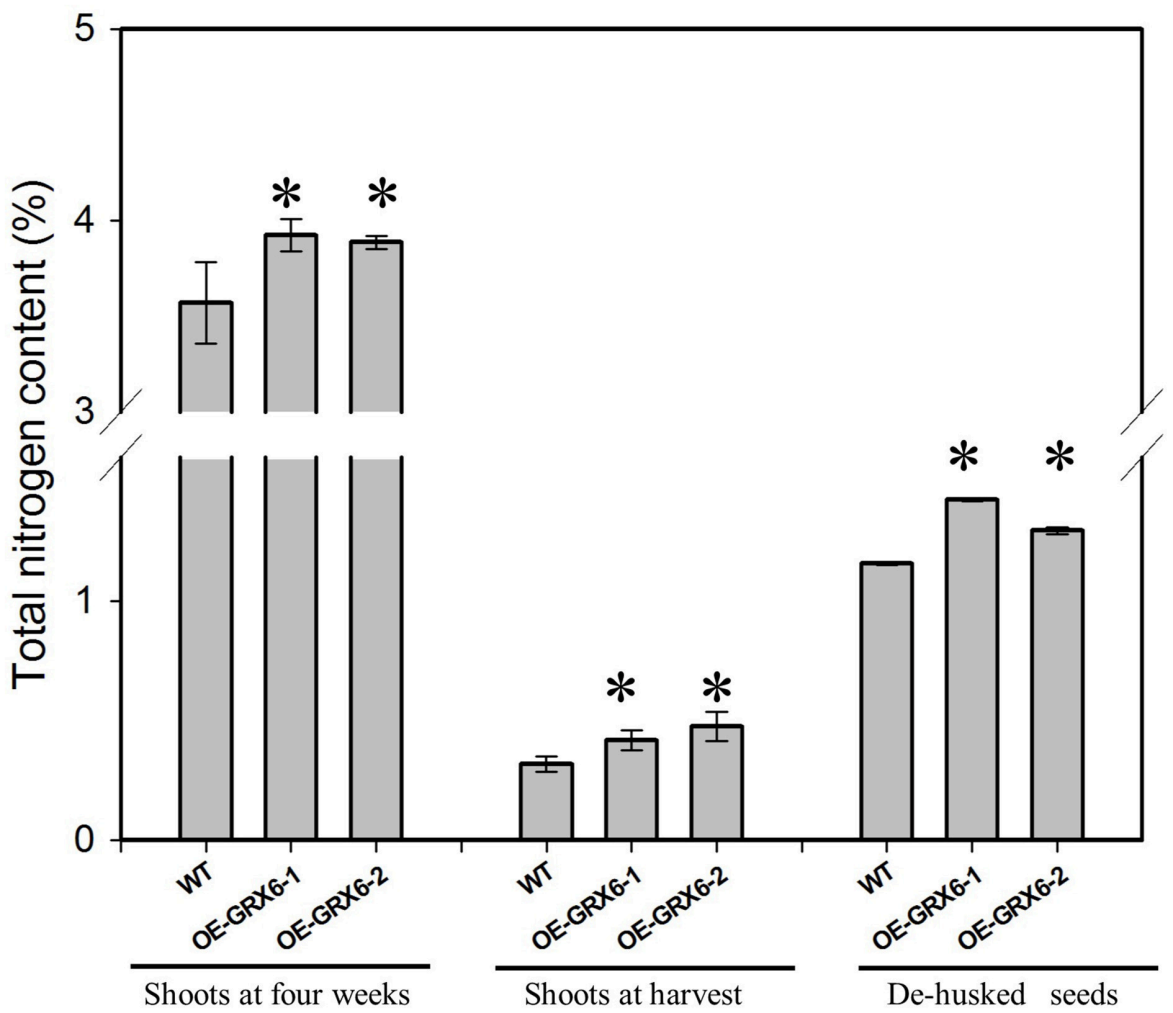
**Total nitrogen content in different plant tissues of transgenic and wild type plants**. When grown in a full nutrient containing medium, transgenic OsGRX6 plants had significantly higher total nitrogen content in the leaves, shoots, and seeds than that of wild type plants. First two groups represent the total nitrogen in the shoots of wild type and transgenic plants collected either at 4 weeks after planting or at harvest. The last group presents the nitrogen content in de-husked seeds. Data are means ±SD of four replicates. Asterisks indicate significant differences at *P* ≤ 0.05 level for each group.

Overexpression of *OsGRX6* resulted in a significant increase in the total nitrogen content; this effect was observed in the shoots of 4 weeks old plants and also at the harvest stage, including in the seeds (Figure 4). However, transgenic plants were found to behave similarly to the wild type under nitrogen limitation (Figure S2).

### Influence of OsGRX6 on chlorophyll degradation and senescence

**Figure 5:**
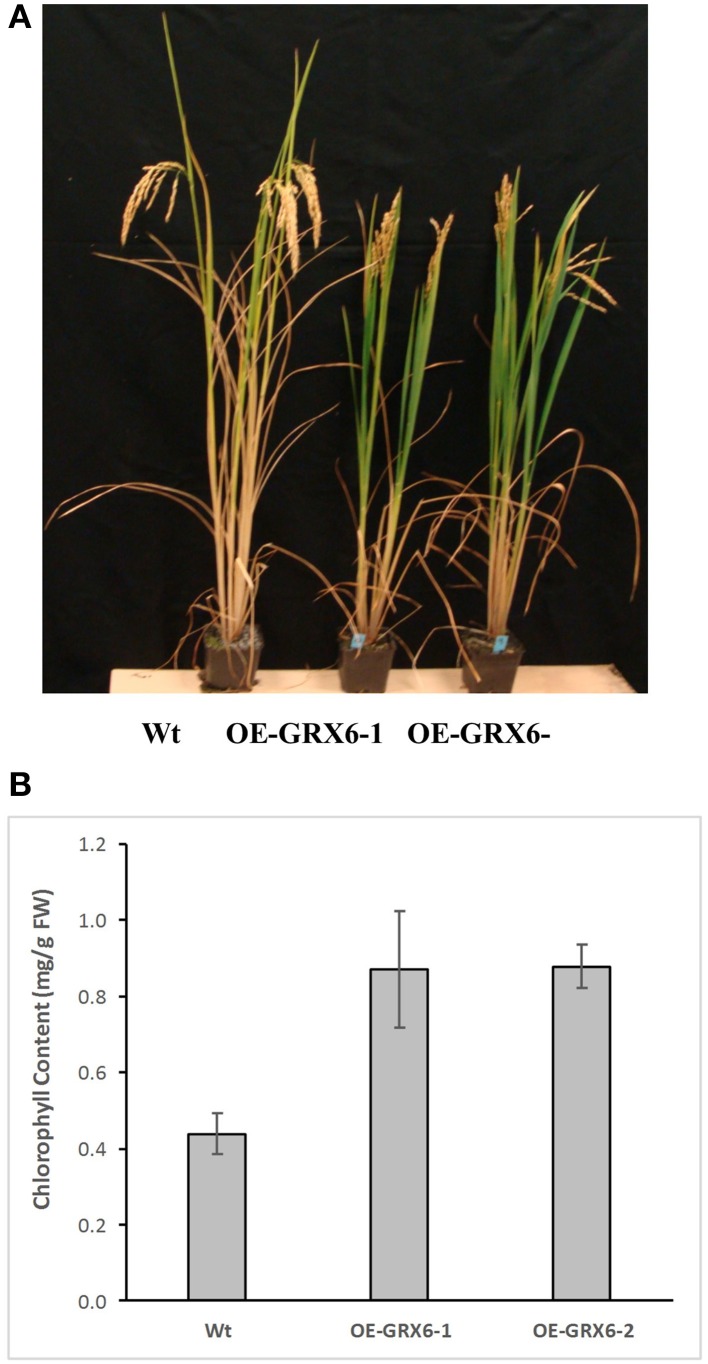
**Chlorophyll degradation in the wild type and transgenic rice lines overexpressed Os GRX6 gene (A) and total chlorophyll content of Wt and OE-GRX6-1 and OE-GRX6-2 (B), 16 weeks after planting**. Data are means ±SD of 4 replicates, each replicate was collected from three different plants.

**Figure 6:**
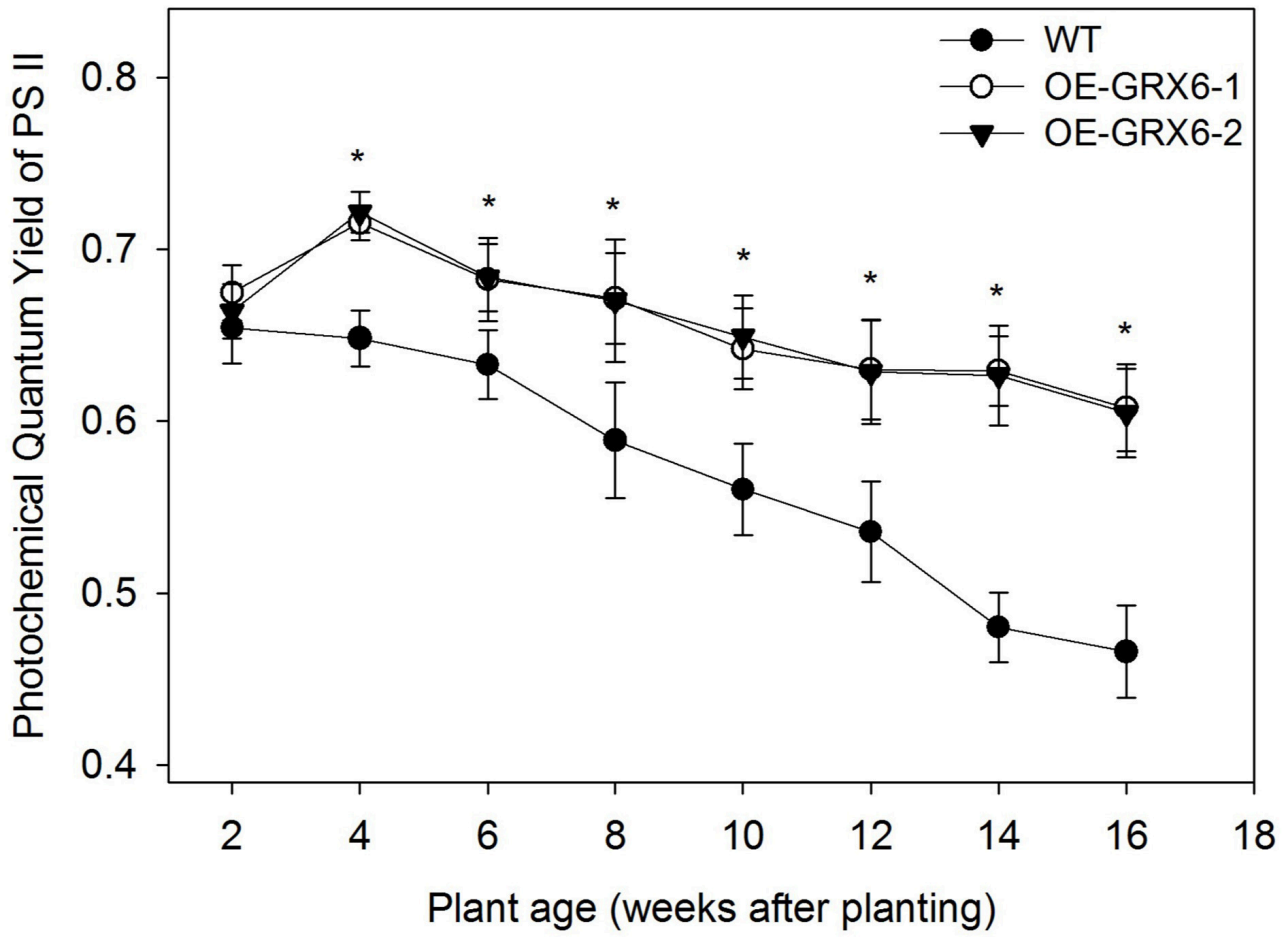
**Photochemical quantum yield of Photosystem II (PS II) of OsGRX6 transgenic and wild type plants measured using Pulse-Amplitude Modulated (PAM) photometry**. There is a significant increase in the Photosystem II activity or photochemical quantum yield of plants in both transgenic lines compared to the wild type at all stages of growth. Data are means ±SD of four replicates. Asterisks indicate significant differences at *P* ≤ 0.05 level for each growth stage.

During the course of this study, we observed that the OsGRX6 overexpressing rice plants had a greener phenotype than the wild-type, even after the fourth week of their growth. This was likely due to greater chlorophyll content and delay in chlorophyll degradation. To confirm this observation, total chlorophyll content was measured in the leaves of wild type and transgenic plants. Total chlorophyll content was significantly higher in both transgenic lines throughout the plant growth cycle than that of wild type plants (Figure 5). Interestingly, the transgenic plants showed a slower level of chlorophyll degradation toward the senescence stage. The observed higher chlorophyll and nitrogen content led us to determine the photosynthetic efficiency of the OsGRX6 transgenic plants compared to the wild type plants. Our results clearly showed that transgenic plants had significantly greater Photosystem II activity/efficiency than wild type plants at all stages of growth (Figure 6).

### *OsGRX6*-overexpressing plants are less sensitive to GA

**Figure 7:**
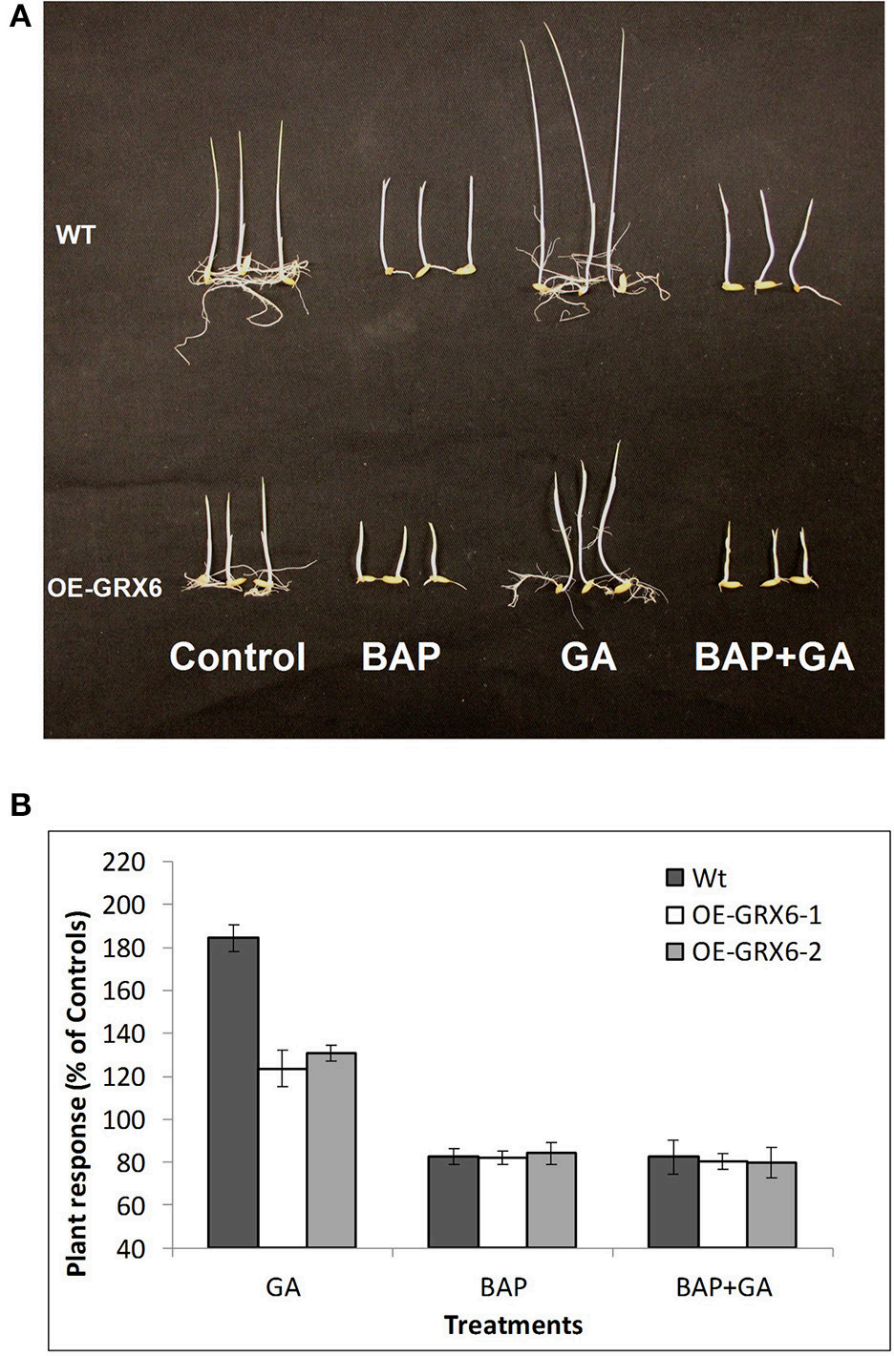
**Effect of exogenous cytokinin and gibberellin on the plant growth**. Wild-type and transgenic seeds were incubated in the nutrient solution supplemented with 100 μ of GA_3_ or/and BAP (6-Benzylaminopurine) for 7 days in the dark at 28°C **(A)**. Response of the Wild-type and transgenic plants to different treatments relative to the untreated controls **(B)**. Data is a mean of 20 seedlings (± SD).

The plant hormones, GA and CK, play a vital role during the plant life cycle. Alteration of GA and/or CK biosynthesis or signaling pathways usually affects plant growth and development. In the present study, we hypothesized that the semi-dwarf phenotype may be due to an alteration in the biosynthesis and/or signaling pathways of either GA or CK. To test this hypothesis, seeds of the wild-type and transgenic plants were grown in the presence of GA and/or 6-Benzylaminopurine (BAP; as a source for CK) for 1 week. Even at this early stage, *OsGRX6* transgenic plants had a shorter and smaller phenotype than the wild type plants (Figure 7). The GA treatment markedly enhanced shoot elongation of wild type plants to reach around 183.9% of the untreated wild type plants (Figures 7A,B). Similarly, *OsGRX6* transgenic plants also increased shoot elongation in response to the GA application, but this increase was much less than that observed in the wild type plants reaching, 123.7% and 130.2% of the untreated OsGRX6 overexpression line 1 and 2, respectively (Figures 7A,B).

In contrast to GA, the CK treatment inhibited plant growth with a reduced size of wild type plants to reach around 83.1% of the control, similarly, the plant height of the transgenic lines treated with CK was reduced to 81.9 and 83.9% of the untreated plants (Figures 7A,B). Interestingly, wild-type plants growing in the presence of CK did not respond to GA treatments, suggesting that CK blocks the GA response on plant growth.

### OsGRX6 alters the hormonal content of the rice plants

**Figure 8:**
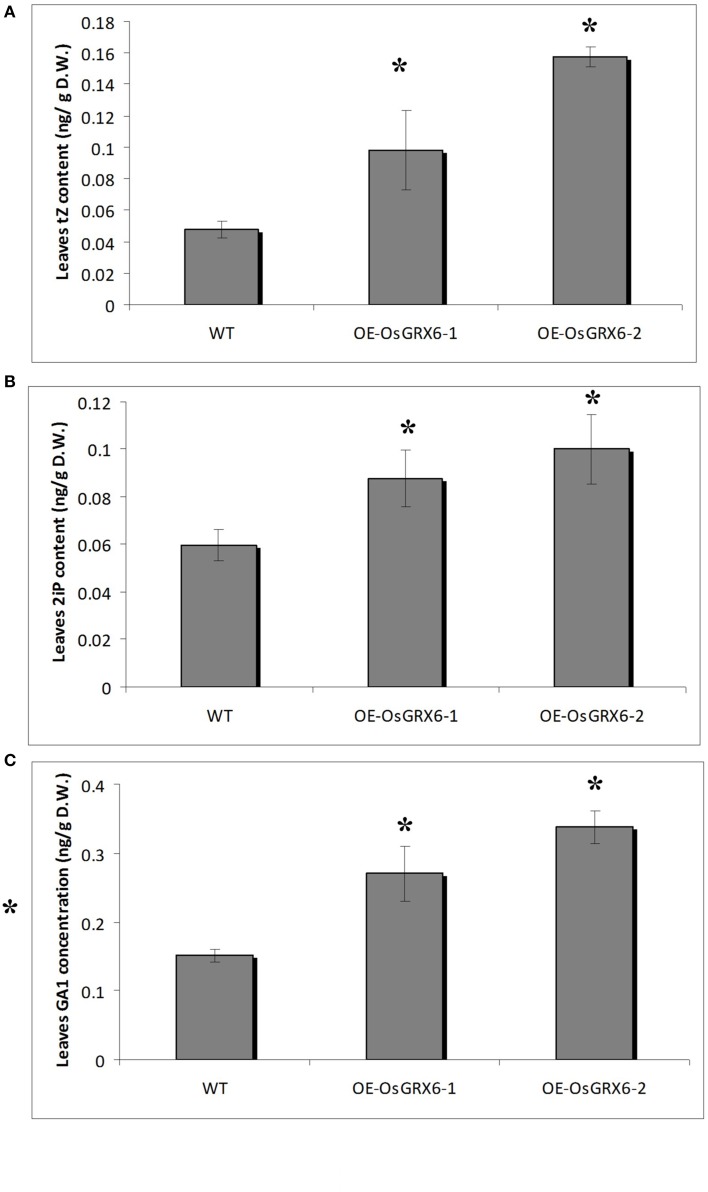
**Cytokinin and GA1 content in rice plants**. The contents of trans-zeatin **(A)**. N6− (Δ2-isopentenyl) adenine **(B)** and the major bioactive GA1 **(C)** in the leaves of 4 weeks old wild-type and transgenic plants growing in the full nutrient medium as described in the materials and method section. Data is a mean of three replicates (± SD). Asterisks indicate significant differences at *p* ≤ 0.05 level.

Hormonal analysis of the OsGRX6 overexpression plants revealed an alteration in the GA and CK contents of the rice plants compared to the wild-type plants. For instance, the leaves of 4 weeks old transgenic rice plants contained significantly higher level of CK compared to the wild type. The contents of *trans*–zeatin (tZ) were 0.1 (±0.03) and 0.16 (±0.01) ng/gFW in the two transgenic lines compared to 0.05 (±0.01) ng/gFW in the wild-type plants (Figure 8A). Consistently, the N^6^- (Δ^2^-isopentenyl) adenine (2iP) reached 0.09 (±0.01) and 0.10 (±0.01) ng/gFW in the transgenic lines compared to 0.06 (±0.01) ng/gFW in the wild-type plants (Figure 8B). Surprisingly, there was a significant increase in the level of the major bioactive GA_1_ was observed in the transgenic plants. The level of GA_1_ was 0.015 (±0.01) ng/gFW in the wild type plants compared to 0.27 (±0.04) and 0.34 (±0.02) ng/gFW in OsGRX6-1 and OsGRX6-2, respectively (Figure 8C).

### OsGRX6-overexpression alters GA and CK biosynthesis and signaling pathways

**Figure 9:**
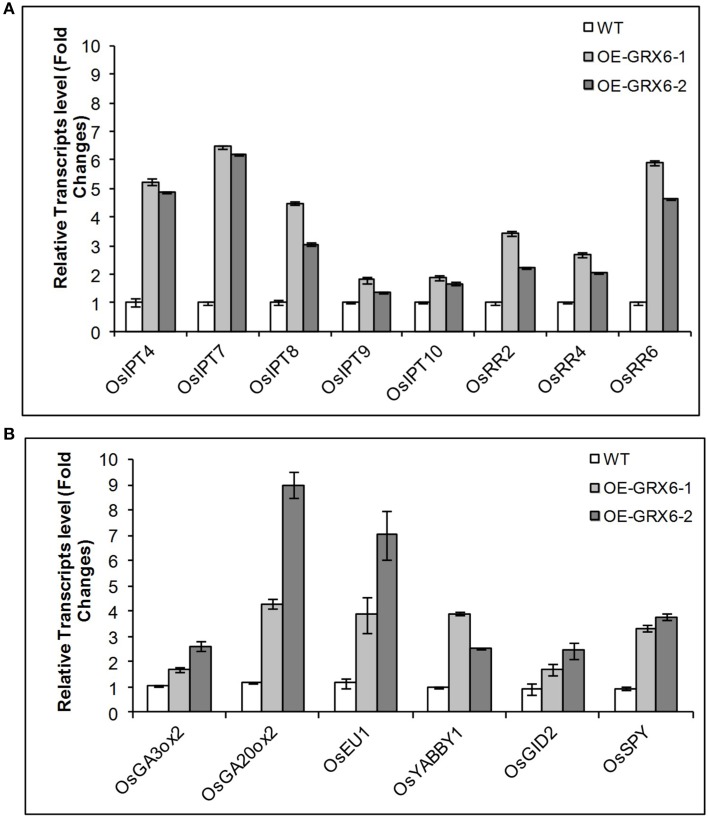
**Quantitative relative gene expression of Cytokinin (A) and GA (B) genes in the wild-type rice and the transgenic lines**. Fully expanded leaves were collected from 4 weeks old plants growing as described in the Materials and Methods. Data represent the values obtained from three biological replicates relative to the wild type plants as a references. Values are the mean of three biological and three technical replicates (±SD).

The alteration in the GA and CK contents observed in OsGRX6 overexpression plants could be due to the modulation in the hormonal biosynthesis or signaling pathways. To examine the two pathways, transcription of a number of genes involved in GA and CK biosynthesis and signaling pathways were examined. Consistent with the hormonal analysis, the *OsGRX6*-overexpressing plants showed higher levels in the transcript level of several genes involved in GA and CK biosynthesis pathways. The quantitative Real time PCR showed an increase in the transcription of the genes involved in CK biosynthesis pathway such as *OsIPTT4, OsIPTT7, OsIPTT8, OsIPTT9*, and *OsIPTT10*. In addition, *OsGRX6* up-regulated the transcription of the genes involved in the CK signaling pathway such as *OsRR2, OsRR4, OsRR6* (Figure 9A). Similarly, the transcript level of *OsGA3ox2* and *OsGA20ox2*, two of the major genes responsible for active GA biosynthesis were up-regulated 2–3 and 4–9 times in the transgenic plants compared to the wild type (Figure 9B). In addition, overexpression of *OsGRX6* also increased the transcript level of the GA receptor *OsGID2* and the GA catabolism gene, *EUI* by 1.5–2.5 and 3–7 folds, respectively (Figure 9B).

The up-regulation of both GA and CK biosynthesis pathways indicates that *OsGRX6* might be involved in the cross talk between the two hormones. DELLA protein, the GA negative regulator was reported to be involved in the crosstalk between GA and CK signaling pathways. Although, the *Arabidopsis* genome contains five DELLA proteins, the rice genome contains only one DELLA protein named SLR1 which controls the plant GA responses. To test whether the rice *OsGRX6* is playing its role through DELLA proteins, real time PCR was carried out to measure the level of SLR1 transcript in the transgenic plants overexpressing *OsGRX6*. Results showed the up-regulation of the transcripts of the *SLR1* gene which were 2–3 time higher in the transgenic plants compared to the wild-type plants (Figure 9B). However, in contrast, western blot analysis did not show any increase in the DELLA protein level in 4 days transgenic plants compared to the wild-type plants (Figure S3).

### OsGRX6-overexpression reduces the cell length of the leaf sheath

**Figure 10:**
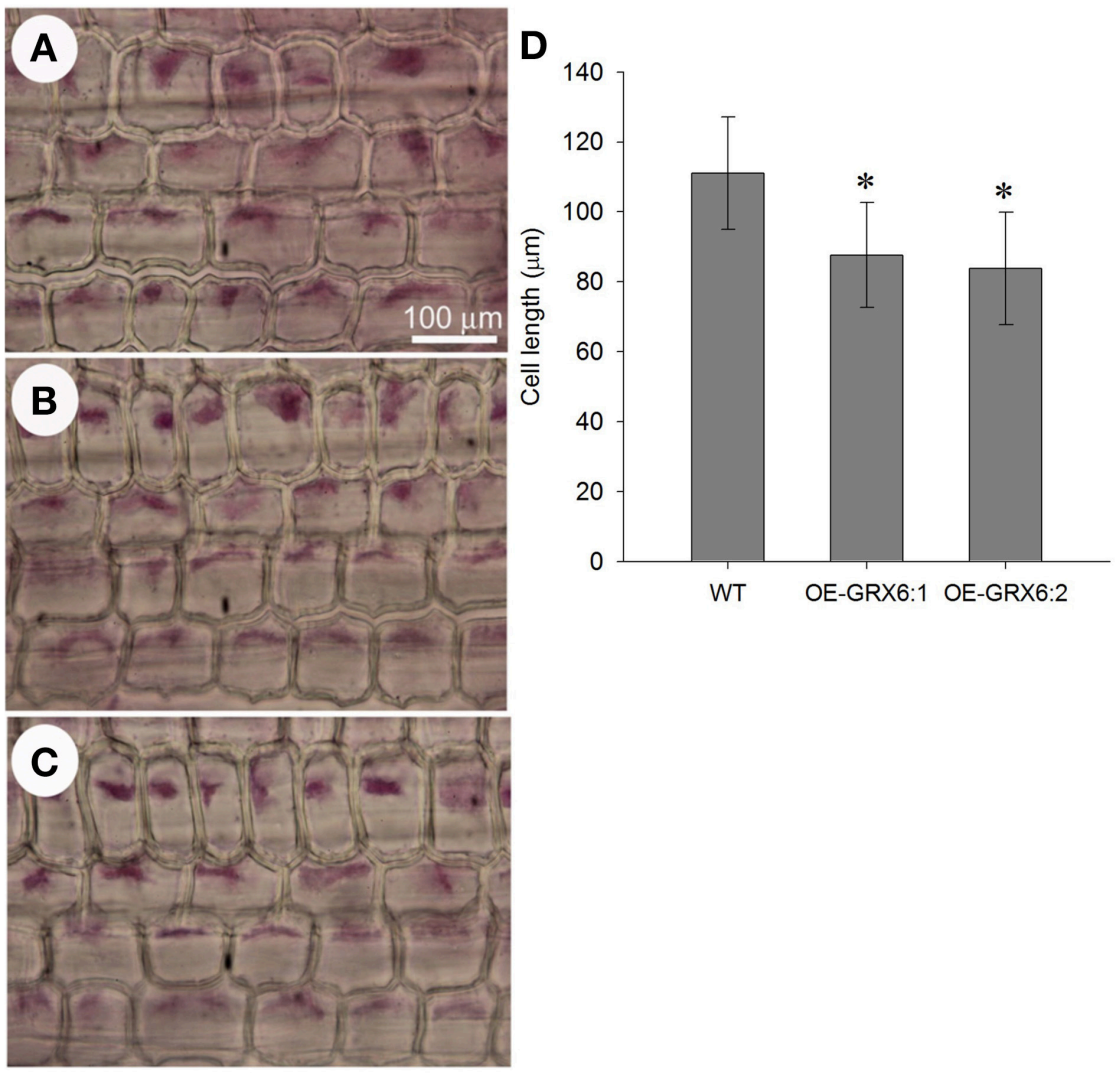
**Leaf sheath cell size in the wild type (A) and in the transgenic lines OE-GRX6-1 and OE-GRX6-2 (B,C) 4 weeks after planting, and cell length (D)**. Data is a mean of 15 measurement (± SD). Asterisks indicate significant differences at *p* ≤ 0.05 level.

The transgenic plants overexpressing OsGRX6 were shorter than the respective wild type plants (Figures 2B,D,E). This was directly correlated with the shorter cell lengths of OsGRX6 overexpressed plants. As summary, the cell length of the leaf sheath of OsGRX6 plants was significantly shorter being ~82% of the length of the wild type plants (Figures 10A–D).

## Discussion

In the present study, we have shown that overexpression of *OsGRX6* in rice affects plant growth and development resulting in a semi-dwarf phenotype. Similar phenotypes have been reported in mutants affecting hormone biosynthesis and signaling pathways, particularly those affecting GA and CK (Sasaki et al., 2003; Kaneko et al., 2004; Thomas and Sun, 2004; Sakamoto et al., 2006). CK and GA levels were analyzed and found to be higher in the transgenic plants suggesting that OsGRX6 is affecting hormone metabolism leading to the semi-dwarf phenotype. CK treatment led to a similar decreased growth response in both the wild-type and transgenic lines. However, while GA treatment led to the expected enhanced hypocotyl growth phenotype in the wild-type lines, a much lower enhanced growth response was seen in the transgenic lines. A number of genes involved with GA and CK metabolism and signaling were up-regulated in the transgenic lines. These include a number of genes involved in CK biosynthesis (*OsIPT4, OsIPT7, OsIPT8, OsIPT9* and *OsIPT10*), CK signaling (*OsRR2, OsRR4, OsRR6*), *GA* biosynthesis (*OsGA3ox2* and *OsGA20ox2*), the GA signaling (*OsGID2*), GA catabolism (*EUI*; Figure 6A) and GA negative regulators (*OsYABBY1* and *OsSPY*).

It is clear that overexpression of OsGRX6 leads to a change in expression of a wide array of genes involved in CK and GA metabolism and sensing which almost certainly in turn leads to the change in growth phenotype. This result could be due to the increase in CK levels. Our result also showed that CK could suppress the GA response in wild-type plants (Figure 7). The increase in the GA_1_ levels is possibly due to the feedback regulation caused by the lower sensitivity to GA, which could be due to the higher CK content in the transgenic plants. Consistent with this notion, OsGA20ox2, an upregulated gene in OsGRX6 overexpressors, was feedback regulated (Ueguchi-Tanaka et al., 2005). The fact that the DELLA level is unchanged in the transgenic plants suggests that this feedback is not directly through DELLA degradation. Instead, it could be through other pathways such as the GA negative regulator OsSPY, which affects GA signaling as well or through other pathways that do not involve the DELLA protein.

These results show for the first time that glutaredoxins are involved in regulating the CK and GA biosynthesis and signaling pathways in plants. Glutoredoxins catalyze the reduction of disulfide bonds of their substrate proteins in the presence of glutathione.

GRX proteins cause a deglutathionylation of a target protein. The catalytic thiol of Grx attacks the disulfide of the glutathionylated peptide, releases the reduced peptide, and becomes glutathionylated. Another molecule of GSH reduces the glutathionylated thiol of Grx (Meyer et al., 2012). One could assume that *OsGRX6* targets one or more glutathionylated proteins involved in GA or/and CK signaling pathway and modifies its function, consequently affecting the plant GA response. However, we still do not know the identity of the OsGRX6 targets.

It seems that OsGRX6 overexpression increases GA and CK biosynthesis through increasing the expression of the biosynthetic genes of *OsIPT-4, OsIPT7, OsIPT8, OsIPT9*, and *OsIPT10* in the CK pathway and of *OsGA3ox2* and *OsGA20ox2* in the GA pathway. In addition, the expression of some negative regulatory genes were up-regulated in the OsGRX6 plants such as the GA negative regulator *OsSPY* which is known for its role in enhancing the CK signaling pathway in *Arabidopsis* (Greenboim-Wainberg et al., 2005). In fact, one reasonable possibility is that OsGRX6 exercises its role through OsSPY.

In addition to the changes in the hormonal biosynthesis and signaling pathways, OsGRX6 overexpression alters the nutritional status of rice plants with a significant increase in total nitrogen content in the shoots and the seeds. The simplest possibility is that this could be due to the higher CK content (Singh et al., 2002; Criado et al., 2009). For example, Hirose et al. (2007), reported that increasing CK content by overexpression of *OsRR6* gene affected several transporters in plants. However, we cannot exclude the possibility that the contrary was true and that up-regulation of the CK biosynthesis could be a result of the increasing in nitrogen availability in the OsGRX6 transgenic plants as previously reviewed by Sakakibara (2003). It is well known that CK delays senescence in different plant species and this finding is correlated with what we found in this study that, transgenic plant showed a higher level of CK and a delay in senescence. The delay in senescence might have directly linked to a slower chlorophyll degradation but greater Photosystem II activity/efficiency of OsGRX6 transgenic plants at different stages of the growth period.

Plant glutaredoxins are also involved in several responses, as described in the Introduction Section. Thus, it is also possible that OsGRX6 may have more than one target affecting several pathways independently.

### Conflict of interest statement

The authors declare that the research was conducted in the absence of any commercial or financial relationships that could be construed as a potential conflict of interest.
