# Supplementary material for: Overexpression of the CC-type glutaredoxin, OsGRX6 affects hormone and nitrogen status in rice plants
Source: Front Plant Sci. 2015 Nov 3;6:934. doi: 10.3389/fpls.2015.00934 (PMC4630655; doi:10.3389/fpls.2015.00934)
Supplement: Supplementary file 1 [file Image1.PDF]

A

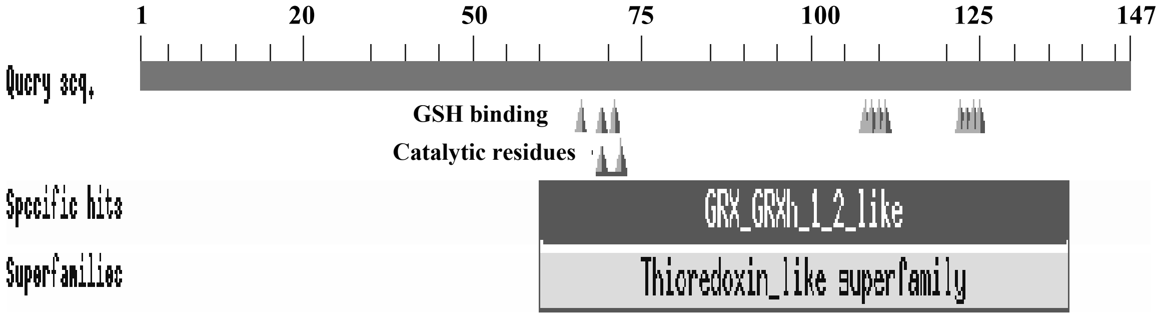

B

1 M Y Q A I P Y S S T R P W L R P E P A A

1 ATGTACCAGGCGATCCCGTACAGCAGCACCCGGCCGTGGCTCAGGCCGGAGCCGGCGGCG

21 S V V D V V K V E T T T A V A G R G G E

61 AGCGTGGTCGACGTCGTGAAGGTGGAGACGACGACGGCCGTCGCGGGTCGGGGCGGTGAG

41 A E V V G E E E A A E V R R A V A E S P

121 GCGGAGGTCGTGGGGGAGGAGGAGGCGGCGGAGGTGCGGAGGGCGGTGGCGGAGAGCCCG

61 V L V V G R R G <u>**C C L I**</u> H V V K R L L Q

181 GTGCTGGTGGTGGGGAGGCGCGGGTGCTGCCTCATCCACGTGGTGAAGCGGCTGCTGCAG

81 G L G V N P A V H E V A G E A A L K G V

241 GGGCTCGGGGTCAACCCGGCCGTGCACGAGGTCGCCGGCGAGGCCGCGCTCAAGGGGGTT

101 V P A G G E A A A L P A V F V G G K L L

301 GTGCCGGCCGGTGGGGAGGCCGCGGCGCTCCCCGCCGTGTTCGTCGGGGGGAAGCTCCTC

121 G G L D R L M A V H I S G E L V P I L K

361 GGCGGGCTCGACCGCCTCATGGCCGTCCACATCTCCGGCGAGCTCGTGCCCATCCTCAAG

141 K A G A L W L *

421 AAGGCCGGTGCCCTCTGGCTTTAA

Figure 1S. Structure and analysis of the OsGRX6 amino cids sequence shows the presence of the Glutaredoxin domain (A) and the CC-Type motif (CCLI) (B).
