# Supplementary material for: Overexpression of the CC-type glutaredoxin, OsGRX6 affects hormone and nitrogen status in rice plants
Source: Front Plant Sci. 2015 Nov 3;6:934. doi: 10.3389/fpls.2015.00934 (PMC4630655; doi:10.3389/fpls.2015.00934)
Supplement: Supplementary file 2 [file Image2.PDF]

A

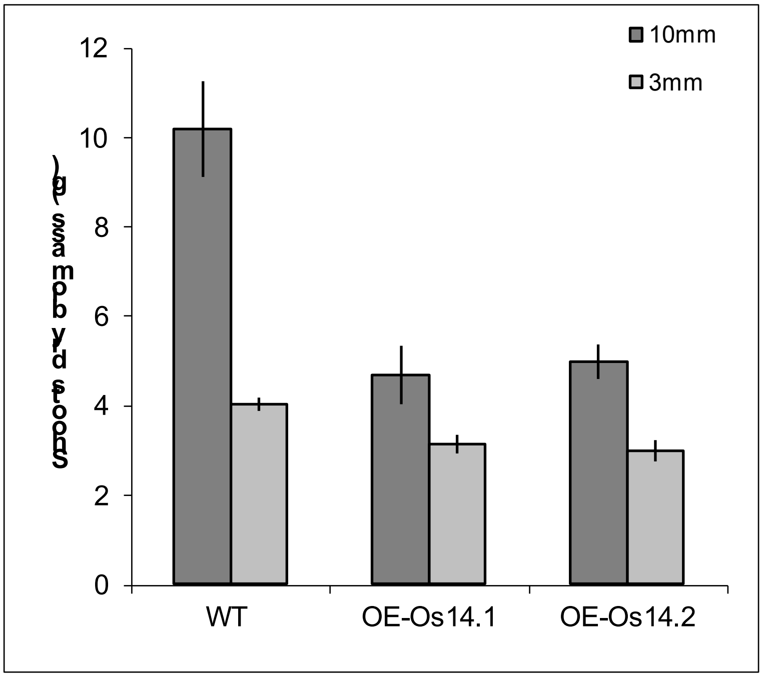

B

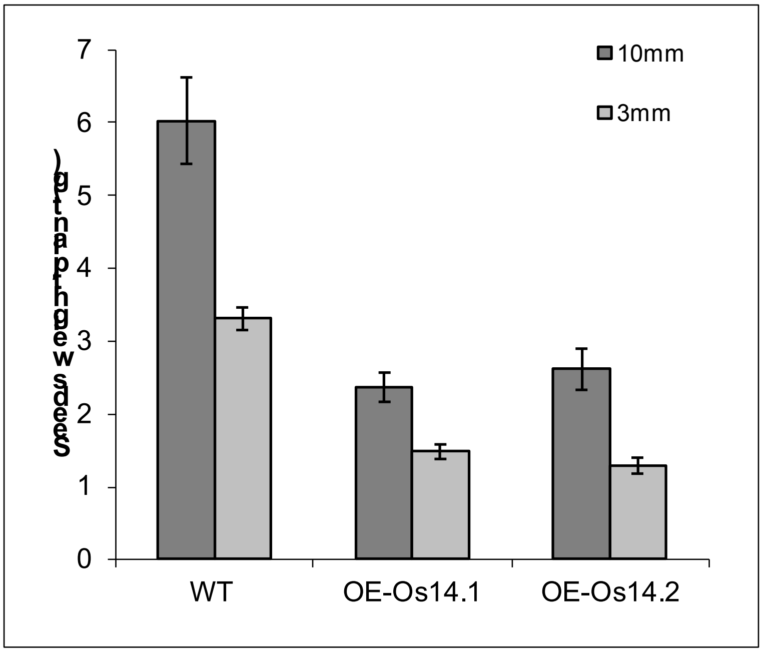

Figure S2. Response of the wild-type and OsGRX6 transgenic plants to sufficient (10mm) and limiting (3mm) nitrogen conditions. Data are means ±SD of 24 plants. Experiments were carried out as described in Bi et al. 2009.
