# Supplementary material for: Overexpression of the CC-type glutaredoxin, OsGRX6 affects hormone and nitrogen status in rice plants
Source: Front Plant Sci. 2015 Nov 3;6:934. doi: 10.3389/fpls.2015.00934 (PMC4630655; doi:10.3389/fpls.2015.00934)
Supplement: Supplementary file 3 [file Image3.PDF]

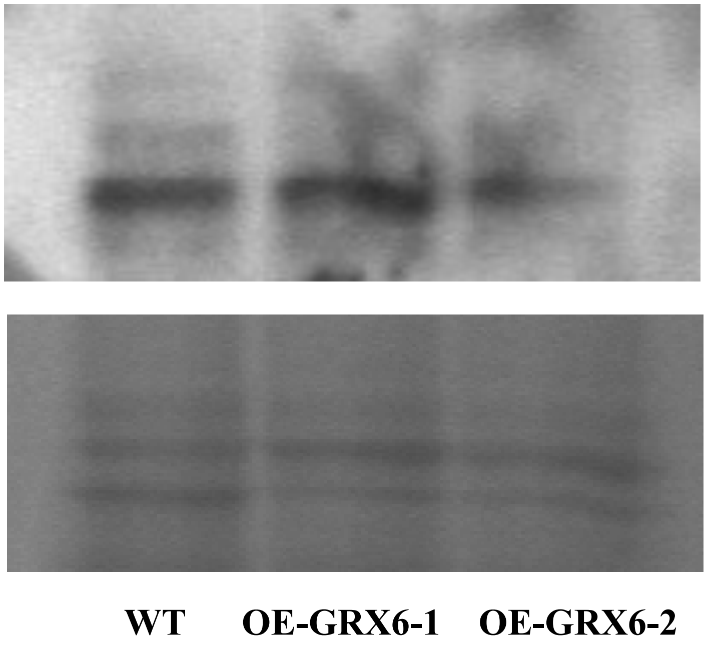

Figure S3. SRL1 level in the wild-type and transgenic plants over-expressed -OsGRX6, one week after germination, lower panel show the commaise blue stain of the total protein.
