# Supplementary material for: Overexpression of the CC-type glutaredoxin, OsGRX6 affects hormone and nitrogen status in rice plants
Source: Front Plant Sci. 2015 Nov 3;6:934. doi: 10.3389/fpls.2015.00934 (PMC4630655; doi:10.3389/fpls.2015.00934)
Supplement: Supplementary file 4 [file Table1.PDF]

Table 1S. Primers used in the QRT-PCR.

Gene	Accession Number	Primer Name	Primer Sequence (5'-3')
OsGRX6	Os01g0667900	OsGRX6F	TCGTGAAGGTGGAGACGA
		OsGRX6R	CGTGGATGAGGCAGCAC
OsIPT4	AB239800	OsIPT4F	AGCGGTACGAGTGCTGCTTC
		OsIPT4R	GGAGCACCTCGAGATCTACGTC
OsIPT7	AB239804	OsIPT7F	AAGATTCAGCTCTATGACGGCC
		OsIPT7R	GAGGCTCACCTTGTTGGTGG
OsIPT8	AB239805	OsIPT8F	ACAAGATTCAGCTGTACGACGG
		OsIPT8R	GCGACACCTTGTTCGTGGT
OsIPT9	AB239806	OsIPT9F	AGGCCTGTGATAACCGGGTA
		OsIPT9R	TGTGTTGCTCCCACTCATGC
OsIPT10	AB239807	OsIPT10F	TCAATCGATCTGAGGTGCGA
		OsIPT10R	GAGTAGGCCACCTGTGTCGG
OsRR2	BR000312	OsRR2F	TACTGCATGCCGGAGATGAC
		OsRR2R	CGCCTTGATGGCTTTGAGAA
OsRR4	BR000258	OsRR4F	CCACTGTGGATTCTGGGAGC
		OsRR4R	CATCCCTCAATCCAAGCAGC
OsRR6	BR000315	OsRR6F	GCGCGTCAAGGAATCGTCT
		OsRR6R	GACATGATCACCACCGGGAT
OsGA3ox2	EU179397	OsGA3ox2F	TTCTCCAAGCTCATGTGGTCC
		OsGA3ox2R	TGAAACTCCTCCATCACGTCAC
OsGA20ox2	AY114310	OsGA20ox2F	TGTCGCTGACGATCATGGA
		OsGA20ox2R	AGTAGTTGCACCGCATGATTG
OsEUI	Os05g0482400	Real2EU1F	ACAAGTGGAGGAAAGCCTACGG
		Real2EU1R	AAGAGTGGCTCCTGGCCTTTCT
OsYABBY	EU846982	OsYABBY1aF	CTTGCTCCTTTTCACCAAGC
		OsYABBY1aR	ATGAGCCCAGTTCTTTGCAG
OsGID2	AB100246	OsGID2F	TTCAGCTCTCGCTGTCACTGTTCT
		OsGID2R	CCCTCCATTCTTATCACTGTCATTTCCC
OsSPY	Os08g0559300	OsSPY1F	CCTCCAGGCACAGAGCCTAC
		OsSPY1R	GCCTCGTTGAAGCACTCGAT
OsActin2	Os10g0510000	OsActin2F	TCTTACGGAGGCTCCACTTAAC
		OsActin2R	TCCACTAGCATAGAGGGAAAGC
